# Benefits of Therapeutic Drug Monitoring of Vancomycin: A Systematic Review and Meta-Analysis

**DOI:** 10.1371/journal.pone.0077169

**Published:** 2013-10-18

**Authors:** Zhi-Kang Ye, Hui-Lin Tang, Suo-Di Zhai

**Affiliations:** 1 Department of Pharmacy, Peking University Third Hospital, Beijing, China; 2 Department of Pharmacy Administration and Clinical Pharmacy, School of Pharmaceutical Sciences, Peking University Health Science Center, Beijing, China; Universidad de Valladolid, Spain

## Abstract

**Background and Objective:**

The necessity of therapeutic drug monitoring (TDM) for vancomycin is controversial. The objective of the current review was to evaluate the available evidence for the necessity of TDM in patients given vancomycin to treat Gram-positive infections.

**Methods:**

Medline, Embase, Web of Sciences, the Cochrane Library and two Chinese literature databases (CNKI, CBM) were searched. Randomized controlled studies and observational studies that compared the clinical outcomes of TDM groups vs. non-TDM groups were included. Two reviewers independently extracted the data. The primary outcome was clinical efficacy of therapy. Secondary outcomes included vancomycin associated nephrotoxicity, duration of vancomycin therapy, length of hospital stay, and mortality. Meta-analysis was performed using the Mantel-Haenszel fixed effect method (FEM). Odds ratios (ORs) or weighted mean differences (WMD) with 95% confidence intervals (95%CIs) were calculated for categorical and continuous outcomes, respectively.

**Results:**

One randomized controlled trial (RCT) and five cohort studies were included in the meta-analysis. Compared with non-TDM groups, TDM groups had significantly higher rates of clinical efficacy (OR = 2.62, 95%CI 1.34–5.11 P = 0.005) and decreased rates of nephrotoxicity (OR = 0.25, 95%CI 0.13–0.48 P<0.0001). Subgroup analyses showed that TDM group had significantly higher rates of clinical efficacy in both cohort studies subgroup (OR = 3.04, 95%CI 1.34–6.90) and in Asian population subgroup (OR = 3.04, 95%CI 1.34–6.90). TDM group had significantly decreased rates of nephrotoxicity in all subgroup. There was no significant difference in duration of vancomycin therapy (WMD = −0.40, 95%CI −2.83–2.02 P = 0.74) or length of stay (WMD = −1.01, 95%CI −7.51-5.49 P = 0.76) between TDM and non-TDM groups. Subgroup analyses showed there were no differences in duration of vancomycin therapy. Only one study reported mortality rates.

**Conclusions:**

Studies to date show that TDM significantly increases the rate of clinical efficacy and decreases the rate of nephrotoxicity in patients treated with vancomycin.

## Introduction

Vancomycin has been long considered the gold standard therapy for methicillin-resistant Staphylococcus aureus (MRSA) [1]. Due to the fact that the early use of vancomycin was associated with a number of adverse effects, including nephrotoxicity, infusion-related toxicities and possible ototoxicity, therapeutic drug monitoring (TDM) of vancomycin was advocated [2], [3].

However, the practice of routine monitoring serum vancomycin concentrations has been the subject of intense debate for many years [4]–[9]. This controversy has led many hospitals to not monitor vancomycin serum concentrations, especially in developing countries. The consensus review from the American Society of Health-System Pharmacists, the Infectious Diseases Society of America, and the Society of Infectious Diseases Pharmacists, as well as the consensus review from the Japanese Society of Chemotherapy and the Japanese Society of Therapeutic Drug Monitoring all recommended monitoring serum concentrations of vancomycin to minimize nephrotoxicity and maximize efficacy [10], [11]. However, the clinical outcomes associated with vancomycin TDM have not been systematically quantified.

As clinical failure in patients with MRSA infections has been increasingly reported in recent years [12]–[15], higher serum vancomycin concentrations are needed to guarantee clinical efficacy. Many studies have shown that high proportions of patients do not achieve therapeutic target concentrations of vancomycin [16]–[26]. This could decrease the therapeutic antibacterial activity of vancomycin. We hypothesize that vancomycin TDM would allow a greater proportion of patients to achieve therapeutic target concentrations of vancomycin in the serum and increase clinical efficacy.

The objective of this systematic review and meta-analysis was to evaluate the available evidence regarding the benefits of vancomycin TDM in patients treated with vancomycin for Gram-positive infections.

## Methods

### Search Strategy

Published articles and conference abstracts (until March 29, 2013) that reported the clinical outcomes of monitoring vancomycin serum concentrations were identified through computerized literature searches in Pubmed, Embase, the Web of Sciences, the Cochrane Library, and two Chinese literature databases (CNKI, CBM). References of retrieved articles were also searched for additional studies. The search terms were the combination of text free terms and Medical Subject Headings (MeSH) terms as follow:(“vancomycin”MeSH) and (“therapeutic drug monitoring” OR “TDM” OR “drug monitoring” OR “therapeutic monitoring” OR “serum concentration monitoring” OR “therapeutic drug” OR “drug monitoring”MeSH). No restriction on language was applied.

### Selection Criteria

Two reviewers (Z.K.Y and H.L.T) independently searched the literature and examined the relevant studies for further assessment of data. Each reviewer was blinded to the other reviewer in the process of data extraction. In case of disagreement between the two reviewers, a third reviewer (S.D.Z) was consulted. Both randomized controlled trials (RCTs) and observational studies comparing clinical outcomes of TDM and non-TDM in patients treated with vancomycin were eligible. Reviews, editorials, guidelines, case reports, and studies focusing only on pharmacokinetics and pharmacodynamics were excluded

### Data extraction and outcomes

Data was extracted from the identified studies included the author, year of study and publication, country in which the study was conducted, study design, number of patients enrolled, population characteristics (type and etiology of infection), clinical efficacy, overall mortality, nephrotoxicity, duration of vancomycin therapy, and length of hospital stay. The primary outcome of the review was clinical efficacy. This was generally defined in the individual studies as absence of symptoms and signs and successful eradication of the causative pathogens. Secondary outcomes were duration of vancomycin therapy, length of stay, mortality and nephrotoxicity. Nephrotoxicity was defined as a rise in serum creatinine concentration (SCr) of greater than 44 umol/L (0.5 mg/dl) or 50% greater than the baseline during vancomycin therapy.

### Quality appraisal

Two authors (Z.K.Y and H.L.T) independently assessed the quality of the studies. Discrepancies were resolved by discussion or through consultation with the third reviewer (S.D.Z). The following risks of bias in RCTs were assessed, according to the criteria developed by the Cochrane risk of bias tool: random sequence generation and concealment of allocation; blinding of participants and personnel; blind assessment of outcomes; incomplete outcomes data; selective outcome reporting and other bias. The quality of observational studies was assessed using the NewCastle-Ottawa scales [27].

### Statistical analysis

All statistical analyses were conducted in STATA 12.0. Pooled odds ratios (ORs) and 95% confidence intervals (CIs) were calculated for categorical outcomes (clinical efficacy and nephrotoxicity) using the Mantel-Haenszel fixed-effects models because there was no evidence of significant heterogeneity for these outcomes. Pooled weighted mean differences (WMD) and 95% confidence intervals (CIs) were calculated for continuous outcomes (duration of vancomycin therapy and length of hospital stay) using inverse variance fixed effects models if no significant heterogeneity was present. Otherwise random effects models were used. Subgroup analyses were conducted according to the study design (RCT or Cohort studies) and geographic location of patients (Asian population or non-Asian population). Heterogeneity among studies was assessed using X^2^ test for heterogeneity and quantified using the Higgins I^2^. To account for the low statistical power of the X^2^ test for heterogeneity, P<0.1 was considered significant. If the P<0.1 or I^2^ more than 50%, a sensitivity analysis was conducted to assess the validity of outcomes. Publications bias was assessed by the Begg funnel plot and the Egger's test [28], [29].

## Results

**Figure 1:**
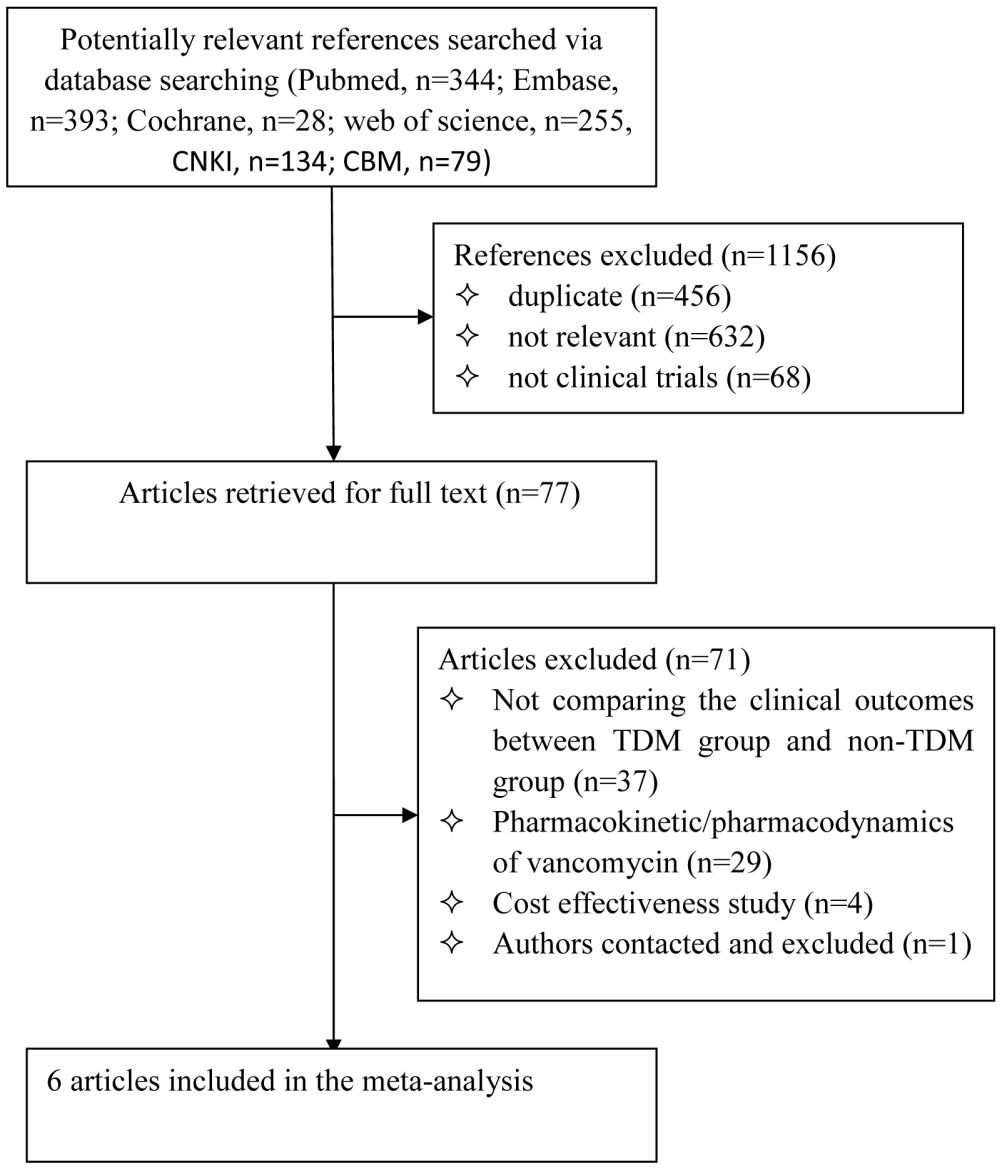
Flow chart depicting the selection process of studies included in the meta-analysis.

Figure 1 shows the study selection process for inclusion in the meta-analysis. We initially identified 1233 potentially relevant studies. A total of 1156 were excluded after review of the titles: 456 were duplicate articles, 632 were not relevant and 68 were not clinical trials or cohort studies. The full-text articles of the remaining 77 studies were evaluated. Another 71 studies were excluded because they did not meet inclusion criteria. Among these, 37 of the studies did not compare the clinical outcomes between the TDM group and the non-TDM group, 29 studies focused only on the pharmacokinetics or pharmacodynamics of vancomycin (n = 29), 4 studies were cost effectiveness studies, and the fulltext could not be obtained from 1 study even after contacting the corresponding author. Six studies were ultimately included in the meta-analysis [30]–[35]. These studies included a total of 521 patients; 249 managed with TDM and 272 managed without TDM.

### Study description

A summary description of the included studies is reported in Table 1. All studies reported on patients treated between 1990 and 2010. Of these, three studies were conducted in Japan, one study in the United States, one study in Spain, and the last in China. Five studies were cohort studies, and one study was a RCT. Welty et al. [30] did not report the type of infection and pathogens. Iwamoto et al. [32] compared the incidence of nephrotoxicity and the duration of vancomycin therapy between the TDM group (n = 77) and the non-TDM group (n = 111), Patients with pneumonia or bacteremia in both the TDM (n = 53) and the non-TDM groups (n = 46) were extracted and compared. In this study, nephrotoxicity was defined as a rise in Scr of greater than 0.3 mg/dl. Nephrotoxicity was classified into three categories: (1) a rise in Scr of greater than 0.3–05 mg/dl; (2) a rise in Scr of greater than 0.6–0.9 mg/dl; (3) a rise in Scr of greater than 1 mg/dl. We excluded patients with a rise in Scr of greater than 0.3–0.5 mg/dl and only included patients with a rise in Scr of greater than 0.5 mg/dl when we conduct statistical analysis about nephrotoxicity. Sato et al. [33] did not report the definition of nephrotoxicity.

**Table 1:** The main characteristics of studies included in the meta-analysis.

Reference	Design, years, country	Setting	Type of infection	Pathogens	Number of patients		Nephrotoxicity definition	Mean age (yr)		Gender (male/female)		Renal function		Weight (kg)	
					TDM	non-TDM		TDM	non-TDM	TDM	non-TDM	TDM	non-TDM	TDM	non-TDM
Welty 1994 [30]	PrC, SC, 1990–1991, United States	Tertiary-care, teaching hospital	NR	NR	61	55	Increase in CREA^a^ of greater than 0.5 mg/dl	58±17	55±19	40/21	32/23	NR	NR	75.7±20.9	70.5±17
Fernandez de Gatta 1996 [31]	RCT, SC, Spain	NR	Empirical treatment, Immunocompromised febrile patients with hematological malignancies with persistent fever more than 72 h	A strong suspicion of infection caused by gram-positive organisms	37	33	Increase in CREA^a^ of greater than 0.5 mg/dl. Mild: 0.5–0.9 Moderate: 1.0–2.4 Severe: greater than 2.5 mg/dl	50.2±18.7	56.3±13.7	29/8	21/12	0.88±0.31(mg/dl)	0.83±0.25 (mg/dl)	62.0±11.2	65.7±12.0
Iwamoto 2003 [32]	ReC, SC, 1996–2002, Japan	NR	Pneumonia, bacteremia and other infections	MRSA	73	111	Increase in CREA^a^ of greater than 0.3 mg/dl. Classify into three categories: greater than 0.3–05 mg/dl; 0.6–0.9 mg/dl and 1 mg/dl	51.8±25.8	53.3±23.1	48/25	73/38	59.6±16.3 (ml/min)	60.4±19.5 (ml/min)	NR	NR
Sato 2007 [33]	PrC, 2003–2006, Japan	NR	All	MRSA	48	31	NR	NR	NR	NR	NR	NR	NR	NR	NR
Mochizuki 2010 [34]	ReC, SC, 2006–2009 Japan,	NR	Catheter related bloodstream infections	CNS	12	8	Increase in CREA^b^ of greater than 0.5 mg/dL or 50% greater than the baseline	53.8±19.6	64.3±7.7	9/3	6/2	0.71±0.26 (mg/dl)	0.72±0.09 (mg/dl)	52.8±12.6	49.5±11.3
Huang 2011 [35]	PrC, SC, 2007–2010 China,	NR	Bloodstream infection, pneumonia, urinary tract infection, Skin and soft tissue infection	MRSA, MRCNS or enterococcus	18	34	Increase in CREA^c^ of greater than 0.5 mg/dl or 50% greater than the baseline	52±18	51±19	10/8	21/13	86.1±35.2 (umol/L)	83.8±31.9 (umol/L)	64±11	67±10

Pr: prospective; C: cohort; Re: retrospective; RCT: randomized controlled trials; SC: single-center; CNS: coagulase negative Staphylococcus; NR: not reported; CREA: serum creatinine;

a a rise during vancomycin therapy.

b a rise was found more than two consecutive times after 3 days of vancomycin therapy.

C a rise found more than two consecutive times after several days of vancomycin therapy.

### Quality of included studies

The method used to generate the allocation sequence in the RCT was considered adequate, whereas allocation concealment was not described. Baseline measurements and characteristics were provided and were similar between TDM and non-TDM groups. Withdrawn patients were reported. Evaluation of the study subjects was by an independent investigator who was blinded to their group assignment.

Table 2 shows the quality appraisal of included cohort studies. Two studies were adequate in 8 of the 9 factors assessed, and three were adequate in 6 of the 9. None of the studies completely accounted for the cohorts.

**Table 2:** Quality appraisal of observational studies (indicators from New-Castle-Ottawa scale).

	Quality indicators								
References	1^a^	2^b^	3^c^	4^d^	5A^e^	5B^f^	6^g^	7^h^	8^i^
Welty 1994 [30]	selected group	Yes	Yes	Yes	No	yes	yes	yes	NR
Iwamot 2003 [32]	yes	Yes	Yes	Yes	Yes	Yes	yes	yes	NR
Sato 2007 [33]	yes	Yes	Yes	Yes	No	No	yes	yes	NR
Mochizuki 2010 [34]	selected group	Yes	Yes	Yes	No	Yes	yes	yes	NR
Huang 2011 [35]	Yes	Yes	Yes	Yes	Yes	Yes	yes	yes	NR

NR, not reported.

a Indicates exposed cohort truly representative.

b Non-exposed cohort drawn from the same community.

c Ascertainment of exposure from a secure record.

d Outcome of interest not present at start of study.

e Cohorts comparable on basis of site and etiology of infection.

f Cohorts comparable on other factors.

g Assessment of outcome of record linkage or independent blind assessment.

h Follow-up long enough for outcomes to occur.

i Complete accounting for cohorts.

### Primary outcomes

#### Clinical efficacy

**Figure 2:**
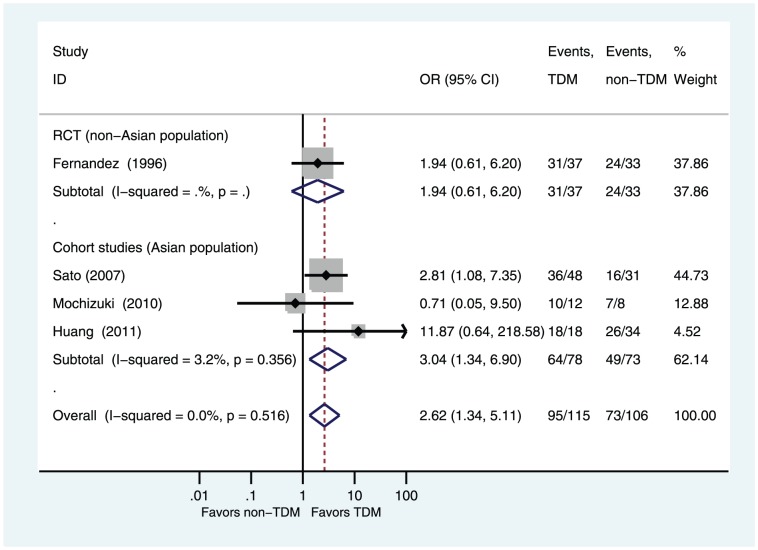
Odds ratios of clinical efficacy: TDM versus non-TDM. Test of clinical efficacy for overall effect: Z = 2.82 P = 0.005; test of clinical efficacy in cohort studies for overall effect: Z = 2.65 P = 0.008; test of clinical efficacy in RCT for overall effect: Z = 1.12 P = 0.265.

Four studies reported the primary outcome: rates of clinical efficacy [31], [33]–[35]. Compared with the non-TDM group, the TDM group had a significantly higher rate of clinical efficacy (OR = 2.62, 95%CI 1.34–5.11 P = 0.005; Figure 2). Subgroup analyses showed that TDM group had a significantly higher rate of clinical efficacy in cohort studies subgroup (OR = 3.04, 95%CI 1.34–6.90 P = 0.008; Figure 2) and in Asian population subgroup (OR = 3.04, 95%CI 1.34–6.90 P = 0.008; Figure 2). Only one study in RCT subgroup and in non-Asian population subgroup (OR = 1.94, 95%CI 0.61–6.20, P = 0.26; Figure 2). No significant heterogeneity was found among the studies (I^2^ = 0%, P = 0.52).

### Secondary outcomes

#### Nephrotoxicity

**Figure 3:**
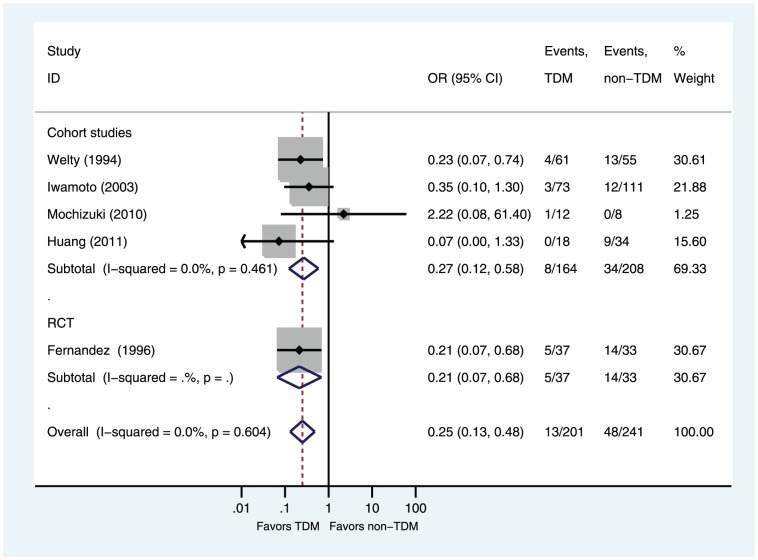
Odds ratios of nephrotoxicity (subgroup analysis by design): TDM versus non-TDM. Test of nephrotoxicity for overall effect: Z = 4.17 P<0.0001; test of nephrotoxicity in cohort studies for overall effect: Z = 3.31 P = 0.001; test of nephrotoxicity in RCT for overall effect: Z = 2.60 P = 0.009.

**Figure 4:**
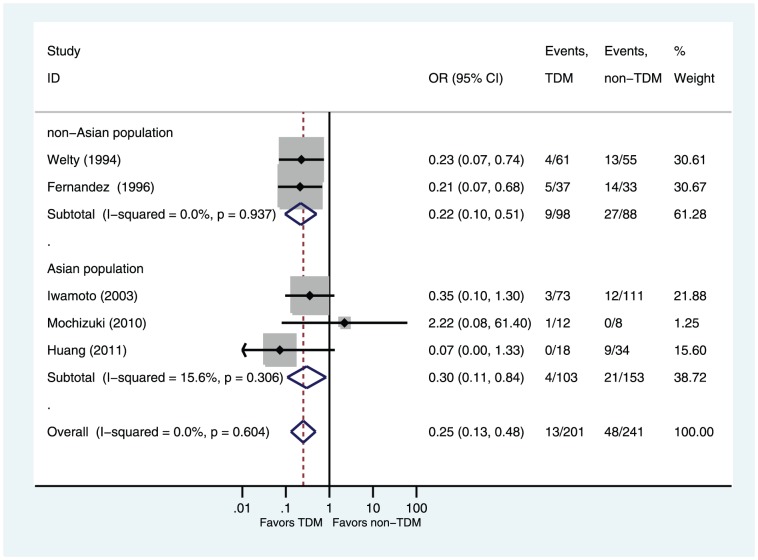
Odds ratios of nephrotoxicity (subgroup analysis by geographic location of patients): TDM versus non-TDM. Test of nephrotoxicity for overall effect: Z = 4.17 P<0.0001; test of nephrotoxicity in Asian population subgroup for overall effect: Z = 2.30 P = 0.022; test of nephrotoxicity in non-Asian population subgroup for overall effect: Z = 3.56 P<0.0001.

Five of six studies reported rates of nephrotoxicity [30]–[32], [34], [35]. Compared with the non-TDM group, the TDM group had a significantly decreased risk of nephrotoxicity (OR = 0.25, 95%CI 0.13–0.48 P<0.0001). Subgroup analyses showed that TDM group had a significantly decreased risk of nephrotoxicity in cohort studies subgroup (OR = 0.27, 95%CI 0.12–0.58 P = 0.001; Figure 3) and in RCT subgroup (OR = 0.21, 95%CI 0.07–0.68 P = 0.009; Figure 3). TDM group had a significantly decreased risk of nephrotoxicity in Asian population subgroup (OR = 0.30, 95%CI 0.11–0.84 P = 0.022; Figure 4) and in non-Asian population subgroup (OR = 0.22, 95%CI 0.10–0.51 P = 0.0004; Figure 4). No significant heterogeneity was found among the studies (I^2^ = 0%, P = 0.60).

#### Duration of vancomycin therapy

**Figure 5:**
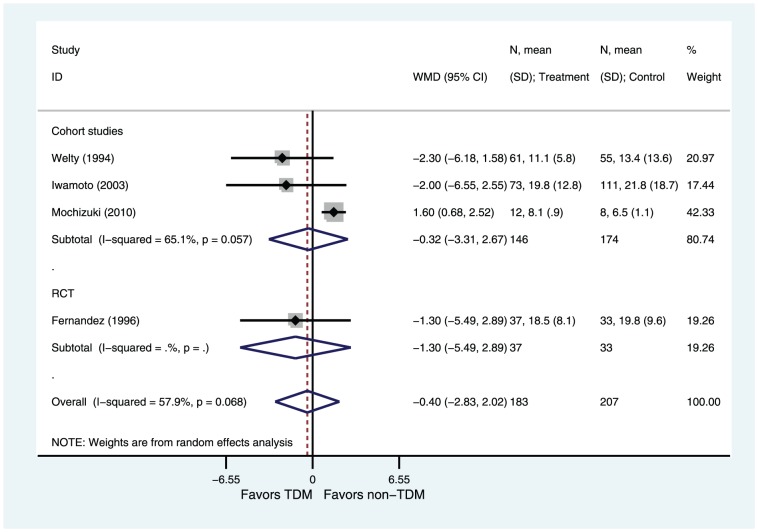
Weight Mean Difference of Duration of vancomycin therapy (subgroup analysis by design): TDM versus non-TDM. Test of duration of vancomycin therapy for overall effect: Z = 0.33 P = 0.74; test of duration of vancomycin therapy in cohort studies for overall effect: Z = 0.21 P = 0.83; test of duration of vancomycin therapy in RCT for overall effect: Z = 0.61 P = 0.54.

**Figure 6:**
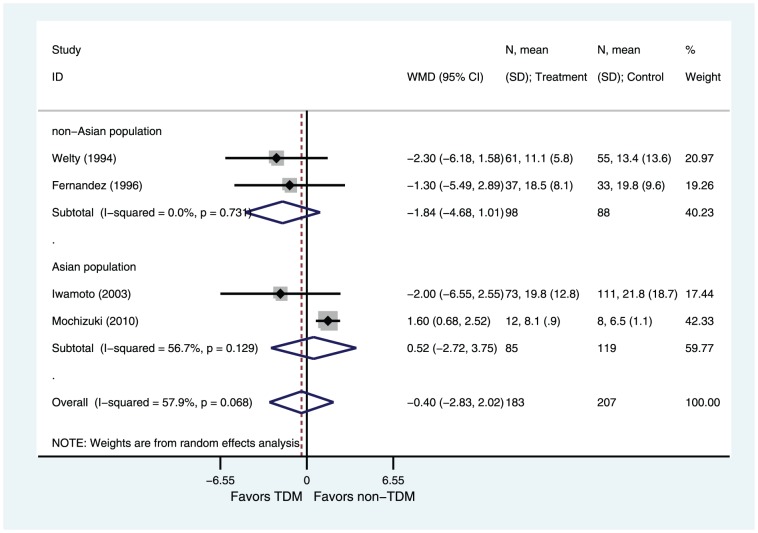
Weight Mean Difference of Duraton of vancomycin therapy (subgroup analysis by geographic location of patients): TDM versus non-TDM. Test of duration of vancomycin therapy for overall effect: Z = 0.33 P = 0.74; test of duration of vancomycin therapy in Asian population subgroup for overall effect: Z = 0.31 P = 0.75; test of duration of vancomycin therapy in non-Asian population subgroup for overall effect: Z = 1.27 P = 0.21.

Four of six studies reported data on the duration of vancomycin therapy [30]–[32], [34]. There was significant heterogeneity among these studies (I^2^ = 58%, P = 0.07). Based on a random effects model, there were no significant differences in the mean duration of therapy in the two group (WMD = −0.40, 95%CI −2.83–2.02 P = 0.74) (Figure 5). Subgroup analyses showed that there were no significant differences in cohort studies subgroup (WMD = −0.32, 95%CI −3.31–2.67 P = 0.83; Figure 5), in RCT subgroup (WMD = −1.30, 95%CI −5.49–2.89 P = 0.54; Figure 5), in Asian population subgroup (WMD = 0.52, 95%CI −2.72–3.75 P = 0.75; Figure 6) and in non-Asian population subgroup (WMD = −1.84, 95%CI −4.68–1.01 P = 0.21; Figure 6). The sensitivity analysis of studies evaluating duration of vancomycin therapy showed a high level of robustness (Figure S1).

#### Length of hospital stay

**Figure 7:**
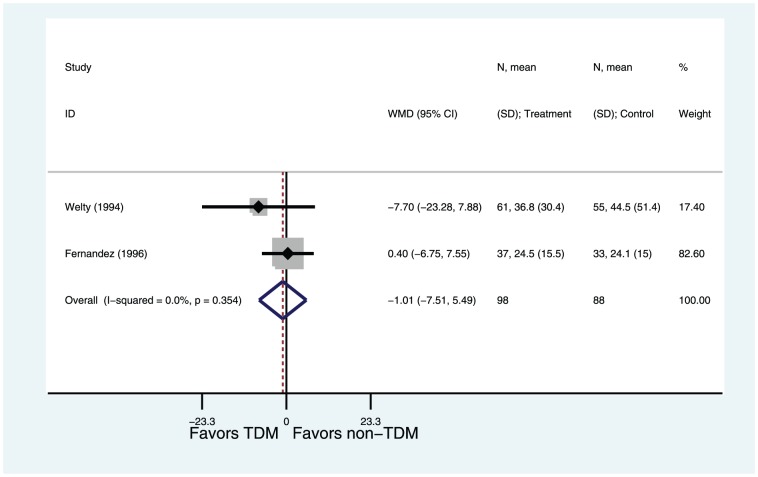
Weight Mean Difference of Length of stay: TDM versus non-TDM. Test of nephrotoxicity for overall effect: Z = 0.30 P = 0.76.

Two of six studies reported length of hospital stay [30], [31]. The length of stay for TDM group was not significantly shorter compared with the non-TDM group (WMD = −1.01, 95%CI −7.51–5.49, P = 0.76). There was no significant heterogeneity between these studies (I^2^ = 0%, P = 0.35) (Figure 7).

#### Mortality

Only one study provided overall mortality rates of the TDM groups and non-TDM groups and showed that two deaths occurred in TDM group (n = 37) in TDM group and six deaths occurred in non-TDM group (n = 33) [31].

### Publication bias

Publication bias were not detected for studies evaluating clinical efficacy (Begg's test P = 1.000, Egger's test P = 0.944), nephrotoxicity (Begg's test P = 0.462, Egger's test P = 0.669) and duration of vancomycin therapy (Begg's test P = 0.308, Egger's test P = 0.130). Studies that evaluated length of hospital stay and mortality rate were inadequate for the assessment of publication bias.

## Discussion

This meta-analysis of prior studies indicates that TDM is associated with significantly higher rates of clinical efficacy and lower rates of nephrotoxicity in patients treated with vancomycin for infections with Gram-positive organisms. Subgroup analyses also showed that TDM is associated with significantly higher rates of clinical efficacy and lower rates of nephrotoxicity. There were not enough data to evaluate the clinical efficacy in RCT subgroup and non-Asian population subgroup. The higher rates of clinical efficacy in the TDM groups is probably due to the fact that a higher proportion of patients had managed with TDM achieved vancomycin serum concentrations in therapeutic range. The lower rate of nephrotoxicity in the TDM is likely due to a lower proportion of patients with supratherapeutic serum concentrations of vancomycin. Vancomycin TDM can significantly decreased the rate of nephrotoxicity in Asian population and non-Asian population, which showed this effect did not dependent on geographic location of patients.

We found no significant differences in the duration of vancomycin therapy in between TDM patients and non-TDM patients, even though there was a tendency to decrease duration of vancomycin therapy. All subgroup analyses also showed that there were no significant differences in between TDM group and non-TDM group. The reason probably could be is that a high proportion of patients still did not reach the therapeutic concentration and the TDM may have started is too late in the TDM group. Iwamoto [32] reported that half of the patients in the TDM group did not reach a therapeutic concentration of vancomycin within 10 days of initial therapy and that it often took more than 10 days to reach therapeutic concentrations. This is detrimental since maximizing the effects of vancomycin is vitally important when treating MRSA infections. The time it takes to achieve the target concentration of vancomycin may be associated with the duration of vancomycin therapy and clinical outcomes [36].

To our knowledge, this is the first meta-analysis that supports vancomycin TDM to increase clinical efficacy and decrease nephrotoxicity. One prior systematic review [37] included two studies and showed no differences in clinical efficacy and nephrotoxicity. We did not assess hospital costs in our studies, although previous research has shown that there is a difference in hospital cost when comparing TDM and non-TDM groups. Darko et al [5] calculated the cost of preventing one vancomycin associated nephrotoxicity episode through TDM to be $8363 for intensive care patients, $5000 for oncology patients and $5564 for patients receiving concomitant nephrotoxins. Similarly, Fernandez de Gatta et al [31] calculated the cost of preventing one mild or moderate vancomycin associated nephrotoxicity to be $435 and $1307, respectively. The results of these two studies indicate that TDM of vancomycin is probably cost-effective, with the biggest potential savings from intensive care patients, oncology patients and patients receiving concomitant nephrotoxins.

Not enough data was available for our meta-analysis to assess which group of patients needed vancomycin TDM the most. However, it should be noted that some special types of patients probably need vancomycin TDM more than others. Some studies have shown wide inter-patient variability in vancomycin pharmacokinetics among adults [38], especially in critically ill patients, patients with severe sepsis, patients undergoing continuous veno-venous hemodialysis, cancer patients, neonatal patients, and patients with severe burn injuries [39]–[46].

We used a comprehensive search strategy to identify the largest number of studies possible. There are also limitations that should be considered when managing our results. First, a relatively small number of studies were included and one is a RCT. However, there was no significant heterogeneity associated with most outcomes, suggesting consistency of results, and most of the studies were adequate in the majority of quality variables evaluated. Second, only one study reported the rates of mortality.

In conclusion, our meta-analysis of prior studies indicate that TDM of vancomycin is associated with higher rates of clinical efficacy and lower rates of nephrotoxicity in patients with Gram-positive organism infections. Based on our findings, we recommend routine monitoring of serum vancomycin concentrations. This may be particular useful for patient at the greatest risk of altered vancomycin pharmacokinetics.

## Supporting Information

Figure S1 **Sensitivity analysis of studies evaluating duration of vancomycin therapy.**
(EPS)

Tabel S1 **PRISMA checklist of this meta-analysis.**
(DOC)
